# Mapping English language teacher resilience: a bibliometric analysis

**DOI:** 10.3389/fpsyg.2026.1790584

**Published:** 2026-04-01

**Authors:** Samet Çağrı Kızkapan, Şule Çelik Korkmaz

**Affiliations:** 1 English Language Teaching Department, Institute of Educational Sciences, Bursa Uludağ University, Bursa, Türkiye; 2 English Language Teaching Department, Faculty of Education, Bursa Uludağ University, Bursa, Türkiye

**Keywords:** bibliometric analysis, English language teacher resilience, multi-database, teacher well-being, thematic evolution

## Abstract

**Introduction:**

Teacher resilience has emerged as a central construct for understanding professional sustainability, well-being, and adaptive functioning in English language teaching (ELT). Although conceptual and empirical interest in ELT teacher resilience has expanded rapidly, existing synthesis work has been limited by single-database coverage and inconsistent validation, constraining cumulative knowledge building and obscuring the field’s intellectual and thematic structure.

**Methods:**

The study employed a multi-database bibliometric mapping design to consolidate and validate research on English language teacher resilience. Bibliographic records were independently retrieved from Web of Science and Scopus using identical search strings and inclusion parameters. Following cross-database validation of temporal, thematic, and geographic robustness, the datasets were merged and de-duplicated, yielding a final corpus of 293 publications. Bibliometric analyses were conducted in R using the bibliometrix package, including performance analysis, co-citation analysis, keyword co-occurrence networks, thematic mapping, and conceptual structure analysis.

**Results:**

Results indicated that ELT teacher resilience research remained marginal until 2018 but entered a phase of rapid expansion from 2021 onward. Knowledge production was concentrated among a small core of authors, journals, and countries, with limited international collaboration in high-output contexts. Co-citation analysis revealed a highly cohesive intellectual structure anchored in positive psychology, teacher emotion research, and adaptive models of professional functioning. Keyword and thematic analyses showed that resilience functioned as an integrative construct embedded within emotional regulation, occupational stress, motivation, and well-being, rather than as an isolated trait. Despite thematic density, constructs such as teacher identity, motivation, and technology use remained weakly integrated.

**Discussion:**

The results position English language teacher resilience as a rapidly consolidating yet structurally imbalanced research domain. While the field demonstrates strong theoretical coherence and psychological grounding, persistent reliance on cross-sectional designs and uneven conceptual integration limit developmental and process-oriented understanding. The study underscores the value of multi-database bibliometric validation and highlights the need for longitudinal, integrative, and theoretically aligned future research in ELT resilience. These results further indicate that teacher education programs and educational authorities should embed structured, developmentally informed resilience support within professional development systems rather than relying on isolated well-being initiatives.

## 1 Introduction

Teacher resilience has increasingly been treated as a consequential construct for sustaining professional functioning and well-being under persistent demands in English language teaching (ELT) context. Resilience has been conceptualized as adaptive skill that supported teachers’ continued effectiveness, commitment, and agency under adversity, including the capacity to “bounce back and thrive rather than just survive” (Ayoobiyan and Rashidi, 2021, p. 293). This conceptualization aligned ELT resilience research with broader psychological research that framed resilience as simultaneously a “process of, capacity for, [and an] outcome of successful adaptation” under challenging circumstances (Masten et al., 1990, p. 426). Within this framing, resilience warranted attention because teachers faced ongoing stressors and shifting institutional expectations, and because adaptation unfolded through both individual resources and contextual affordances rather than through isolated traits (Gu and Day, 2007).

Across resilience studies, conceptual plurality has co-existed with increased precision about mechanisms. Except for Qu et al. (2025) who viewed resilience as a relatively stable personal trait, process-oriented accounts treated adversity as subjectively appraised rather than objectively fixed, which clarified divergent resilience trajectories under similar working conditions through appraisal and reappraisal of stressors and coping options (Schwarze and Wosnitza, 2018). Similarly, social-ecological syntheses also conceptualized resilience as emerging from the interplay of internal and external protective factors rather than being reducible to either domain (Beltman et al., 2011). Complementary models reinforced this dynamic systems view. To clarify, Richardson et al.’s (1990) resiliency model framed adaptation as disruption and reintegration, with “resilient reintegration” supporting growth-oriented adaptation. Likewise, Mansfield et al. (2012) conceptualized teacher resilience as complex, dynamic, and multidimensional, spanning profession-related, emotional, motivational, and social dimensions. Gu (2018) further theorized resilience as a reciprocal developmental process of navigating and negotiating psychological and sociocultural resources across a career. Positive psychology also treated resilience as cultivable through systematic competence-building and strength-based approaches (Seligman and Csikszentmihalyi, 2000), with positive emotions broadening thought-action repertoires and building durable coping resources over time (Fredrickson, 2001).

More specifically, ELT research has adapted these perspectives to the distinctive demands of language teaching, positioning resilience as dynamic, relational, and developmental rather than innate or fixed (Entesari et al., 2020). A consistent body of literature in ELT framed resilience within multilevel models integrating individual, relational, and institutional dimensions. Heng and Chu (2023) characterized resilience as a dynamic process shaped by psychological and environmental-contextual processes, with teachers integrating personal and contextual resources to sustain well-being. Measurement work further suggested that resilience in ELT may require domain-sensitive operationalizations. Accordingly, Shirazizadeh and Abbaszadeh (2023) proposed an ELT-resilience model incorporating motivational, social, emotional, and pedagogical components alongside external supportive factors, while Liu et al. (2024) developed a language teacher resilience scale that included professional, emotional, social, and cultural dimensions. However, studies continued reliance on general resilience instruments (e.g., Entesari et al., 2020; Rezazadeh et al., 2023) indicating a mixed measurement landscape that complicated cross-study comparability and construct-operational alignment at the field level. Empirical work also reinforced a process orientation through links between resilience and reflective and regulatory processes. As a case in point, Ayoobiyan and Rashidi (2021) reported positive associations between reflective practice subscales and resilience, with cognitive and metacognitive reflection predicting resilience, and Zhi and Derakhshan (2024) highlighted positive associations between resilience and psychological well-being as well as resilience and emotion regulation. Despite this pervasive dynamic and developmental framing, much of the empirical evidence operationalized resilience as a largely static attribute captured at a single time point, thereby limiting representations of change over time and obscuring how such tensions are reflected in the aggregate structure of the literature (e.g., Kim and Kim, 2024; Liu, 2024; Wang X. et al., 2024).

Parallel to these conceptual and empirical developments, synthesis work has attempted to consolidate knowledge through bibliometric mapping, yet current evidence has suggested both growth and fragmentation. At the broader field level, Li and Chen (2024) mapped 692 publications (1998–2023) and documented expansion and differentiation, including increased attention to specific contexts such as language teaching. Their country-level mapping highlighted concentration in output and influence alongside uneven citations-per-document patterns across regions, and collaboration networks spanning 66 countries/regions showed instability and many one-off partnerships (Li and Chen, 2024). In the ELT context, Ekizer (2024) synthesized 24 articles (2017–2024) and documented screening from 373 initial records to 24 L2-specific studies, indicating a comparatively thin evidence base sensitive to definitional specificity. With regard to co-citation Gu and Dewaele were positioned as influential nodes and as for source patterns, applied linguistics and teacher education were reported as highlighted journals (Ekizer, 2024). Furthermore, keyword co-occurrence patterns were found to connect resilience to positive psychology, well-being, coping, stress, engagement, and foreign language teaching enjoyment, with “covid” emerging as a newer keyword shaping the research front (Ekizer, 2024).

Despite these advances, review-level literature has revealed persistent gaps that limit cumulative knowledge building and call for more integrative bibliometric validation. Conceptually, existing syntheses have not consistently applied a shared coding framework to distinguish resilience from adjacent protective factors and correlates, increasing the risk of construct drift across reviews (Daniilidou and Pezirkianidis, 2025; Zhang, 2023). Reviews have also rarely imposed a reusable typology for classifying studies by theoretical orientation (e.g., trait-, process-, or context-oriented approaches), constraining cross-review comparability, although Mukadimah (2025) adopted a four-perspective framework within a country-specific scope. Contextually, prior reviews alternated between narrowly bounded national syntheses (Mukadimah, 2025) and large-scale bibliometric mappings constrained by single-database coverage (Li and Chen, 2024), leaving limited coordinated evidence on cross-context regularities versus context-sensitive mechanisms. Methodologically, review practices have varied in transparency and standardization, ranging from PRISMA-aligned bibliometric workflows (Li and Chen, 2024) and structured extraction with adjudication (Daniilidou and Pezirkianidis, 2025) to narrative and mini-review approaches that limited reproducibility. Taken together, these limitations point to the need for multi-database bibliometric validation combined with systematic synthesis procedures that enhance transparency, comparability, and evidentiary robustness.

Beyond extending corpus size, the present study contributes substantively by revealing structural and thematic features of English language teacher resilience research that were not visible in prior syntheses. In contrast to earlier bibliometric mappings conducted either at the macro level of teacher resilience (Li and Chen, 2024) or within narrowly screened ELT-specific subsets (Ekizer, 2024), the present multi-database corpus enables stable identification of conceptual asymmetries within the field. In particular, it exposes the weak integration of theoretically adjacent constructs, such as teacher identity, motivation, and technology-mediated teaching, within the dominant resilience discourse, despite their recognized relevance in teacher psychology. These imbalances are not artifacts of individual databases or retrieval strategies, but persistent structural features of the literature that emerge only after cross-database validation and consolidation.

Accordingly, the present study employed a multi-database bibliometric mapping approach to consolidate English language teacher resilience research and to provide field-level validation of its intellectual and thematic structure. Specifically, the study aimed to answer three bibliometric research questions:

*RQ1*: How has publication on English language teacher resilience evolved over time, and which authors, journals, and countries dominate this domain?

*RQ2*: Based on co-citation analysis, which authors and documents constitute the intellectual structure of English language teacher resilience?

*RQ3*: Based on keyword co-occurrence and thematic evolution, what emerging themes characterize the research front?

## 2 Materials and methods

The bibliometric analysis of the study aimed to map the intellectual, conceptual, and structural landscape of research on English language teacher resilience. Bibliometric analysis was selected because it enables the systematic examination of large bodies of scientific literature, allowing researchers to identify publication trends, influential authors and journals, collaboration patterns, and thematic evolutions across time (Donthu et al., 2021). Consistent with the purpose of bibliometric inquiry, this study was exploratory and descriptive rather than evaluative in nature. The bibliometric analysis was conducted in RStudio using the bibliometrix package to import, clean, and analyze the bibliographic dataset and to generate descriptive performance indicators and science-mapping outputs (e.g., co-authorship, co-citation, and keyword co-occurrence networks) (Aria and Cuccurullo, 2017).

Bibliometric research often relies on multidisciplinary citation databases to ensure comprehensive data coverage and robust validation (Chansanam and Li, 2025). Web of Science (WoS) and Scopus are among the most widely used databases for such analyses due to their extensive coverage and reliability (De Battisti and Salini, 2013). De Battisti and Salini (2013) further emphasize the importance of using multiple bibliometric databases to overcome the limitations of single-source analyses, such as incomplete data coverage and biases. They highlight that Scopus and WoS are leading databases in bibliometric research, with Scopus offering broader journal coverage compared to WoS, which is more selective but widely recognized for its high-quality archival data. Similarly, Chansanam and Li (2025) developed KKU-BiblioMerge, a bibliometric tool specifically designed to integrate data from Scopus and WoS, demonstrating the advantages of multi-database integration in enhancing data inclusivity and accuracy. Their study revealed that combining data from these databases mitigates biases and data gaps, providing a more comprehensive understanding of research landscapes. Bai et al. (2025) further validated the robustness of bibliometric findings through cross-database comparisons between WoS, Scopus, and PubMed, confirming high concordance in publication trends, thematic focuses, and geographical contributions. These studies collectively underscore the critical role of multi-database sourcing in bibliometric research, as it enhances the reliability and generalizability of findings across diverse scientific domains.

Accordingly, the data of this study were retrieved independently from WoS and Scopus, the two most widely used multidisciplinary citation databases in bibliometric research. Using multiple databases was intended to enhance coverage and to support validation through cross-database sourcing. The WoS database was accessed on 4 December 2025 using the All-Fields search option. The Scopus database was accessed on 17 January 2026 using the TITLE-ABS-KEY field to ensure conceptual equivalence with the Web of Science query. In both databases, the researchers applied language filter as English. For Scopus database, the researchers applied year filter to exclude early publications in 2026. The following Boolean search string was applied:

(“resilien*” OR “burnout resistance”) AND (“language teacher*” OR “English teacher*” OR “ESL teacher*” OR “EFL teacher*” OR “ELT teacher*” OR “TESOL teacher*” OR “TEFL teacher*” OR “second language teacher*”*)*

The Boolean search string combined the use of resilien* with the expression burnout resistance using the OR operator to maximize coverage at the retrieval stage. This formulation was adopted to capture the full lexical range through which resilience-related phenomena may be referenced in the literature, particularly in interdisciplinary or early-stage publications. The use of the OR operator reflects a field-mapping orientation rather than an assumption of conceptual equivalence between the two expressions. Throughout the analysis and interpretation, resilience, indexed via resilien*, remained the central organizing construct of the study. Further, an isolated search of the term “burnout resistance” using identical field and temporal restrictions yielded zero records; thus, the term did not contribute to the final corpus.

This search yielded 208 documents from the WoS database, and 213 documents from the Scopus database. To ensure that the bibliometric results were not artifacts of a single indexing platform, a cross-database validation procedure was conducted using WoS and Scopus as independent bibliographic sources. Identical search strings, time spans, and inclusion criteria were applied to both databases, and bibliometric analyses were initially performed separately to assess robustness across sources. Validation focused on three core dimensions commonly used in bibliometric research: temporal publication trends, thematic consistency, and geographic distribution of research output.

First, temporal robustness was examined by comparing annual publication counts derived independently from WoS and Scopus. Pearson correlation analysis revealed an almost perfect positive association between yearly publication trends across databases (*r* = 0.99, *p* < 0.001), indicating that the developmental trajectory of English language teacher resilience research was highly consistent regardless of indexing source. Although absolute publication counts differed between databases, as expected due to variations in coverage, the overall temporal pattern and growth dynamics were strongly aligned.

Second, thematic validation was conducted through a comparison of high-frequency Author Keywords. The top-20 Author Keywords extracted separately from WoS and Scopus demonstrated substantial overlap, with 14 shared keywords corresponding to a 70% convergence rate. The overlapping terms captured the conceptual core of the field, including resilience, EFL teachers, positive psychology, teacher resilience, emotion regulation, self-efficacy, work engagement, well-being, and professional development. Variations were primarily observed among lower-frequency keywords, reflecting database-specific indexing practices rather than substantive thematic divergence.

Third, geographic validation was performed by comparing country-level publication output across databases. Author affiliation data were standardized to single-country attributions to ensure comparability. The leading contributing countries were highly consistent between WoS and Scopus, with China, Iran, the United States, the United Kingdom, Australia, Canada, and several other countries appearing prominently in both datasets. Minor discrepancies emerged only among lower-ranked contributors, consistent with known differences in regional journal coverage across databases. Geographic validation focused on rank convergence and structural consistency rather than absolute publication counts, as country-level quantities are known to vary systematically across bibliographic databases due to differences in indexing scope and affiliation parsing.

Given the strong convergence observed across temporal, thematic, and geographic dimensions, the two databases were subsequently combined for the main bibliometric analysis to maximize coverage and representativeness. This integration was undertaken only after cross-database validation confirmed that the core patterns identified were stable and reproducible across indexing platforms. Accordingly, the final bibliometric mapping and network analyses reported in this study are based on the merged WoS–Scopus dataset.

Cross-database validation was not treated as an assumed strength but as an empirical test of robustness. Independent analyses were first conducted on the Web of Science and Scopus datasets to assess whether temporal trajectories, thematic cores, and geographic distributions converged across indexing platforms. Only after high concordance was observed were the datasets merged for integrative mapping. This procedure ensured that subsequent network structures and thematic configurations reflected stable properties of the field rather than database-specific artifacts. Importantly, convergence across databases does not render multi-database analysis redundant; instead, it provides evidentiary justification for dataset integration while allowing finer-grained structural patterns, including thematic marginality and conceptual under-integration, to be identified with greater confidence.

The two datasets were imported into R and harmonized using the bibliometrix package. Records were retrieved from WoS in plain text export and Scopus in CSV export. Following database integration, duplicate records were identified and removed through a two-stage procedure. First, duplicates were detected via exact DOI matching across databases. Second, for records lacking DOI information, duplicates were identified through exact matching of normalized document title, publication year, and first author. This process resulted in the removal of 128 duplicate records (127 DOI-based and 1 title-based), yielding a final consolidated bibliometric dataset of 293 unique documents. No inclusion or exclusion criteria were applied at this stage. This decision aligned with bibliometric best practices, which recommend minimizing restrictions in order to capture the full scope and heterogeneity of a research field and to avoid prematurely narrowing the intellectual map of the domain (Donthu et al., 2021).

The adequacy of the dataset size was evaluated with reference to Rogers et al. (2020), who demonstrated that samples exceeding approximately 200 publications are sufficient to produce stable and interpretable bibliometric indicators for field-level analyses, particularly when the objective is descriptive mapping rather than institutional ranking or fine-grained citation impact assessment. Accordingly, the corpus of 293 documents was considered methodologically appropriate for generating a reliable overview of the field of English language teacher resilience.

The merged dataset included two keyword fields: Author Keywords (DE) and Keywords Plus (ID). Author Keywords were available for records from both Web of Science and Scopus. Keywords Plus is primarily generated within Web of Science and does not have a direct Scopus equivalent. In the merged corpus (N = 293), Web of Science contributed 208 records (71.0%) and Scopus contributed 85 records (29.0%). Keywords Plus entries were available for 181/208 Web of Science records (87.0%) and 11/85 Scopus records (12.9%). Accordingly, analyses involving Keywords Plus predominantly reflect the Web of Science subset of the corpus.

The corpus consisted of 293 documents spanning a variety of publication formats. To enhance interpretability, document types were grouped into research-oriented publications and non-research or peripheral formats, following common practices in bibliometric reporting.

Research articles constituted the overwhelming majority of the dataset (*n* = 242), supplemented by review articles (*n* = 6) and book chapters (*n* = 17). Together, these formats represented the core scholarly contributions of the field. A small number of books (*n* = 3) were also included.

In contrast, several publication formats reflected editorial, corrective, or dissemination-oriented contributions rather than original empirical research. These included editorials (*n* = 2), errata (*n* = 2), and retracted publications (*n* = 1). Conference-related outputs, such as conference papers (*n* = 4) and proceedings papers (*n* = 1), were minimally represented, indicating that the field’s knowledge production has been primarily disseminated through peer-reviewed journals rather than conference venues.

Although the corpus included editorials, errata, and other non-research formats, these publication types constituted a very small proportion of the dataset. The analysis therefore predominantly reflects trends in peer-reviewed research articles and reviews. To ensure that non-research documents did not influence network topology, a sensitivity analysis was conducted excluding the retracted article (*n* = 1), errata (*n* = 2), and editorials (*n* = 2). Re-analysis yielded substantively identical descriptive and network patterns. Co-citation modularity shifted minimally from 0.124 to 0.126, indicating structural stability (see Table 1).

**Table 1:** Distribution of document types in the corpus.

Category	Document type	*n*
Core research publications	Journal articles	242
	Review articles	6
	Book chapters	17
	Books	3
Conference-related publications	Conference papers	4
	Proceedings papers	1
Editorial and corrective materials	Editorials	2
	Errata	2
	Retracted publications	1
Hybrid/early-stage publications	Early access articles	14
	Article-book chapter hybrids	1

## 3 Results

The final corpus comprised 293 publications retrieved from a merged and deduplicated Scopus and Web of Science dataset, covering the period from 2008 to 2025. These publications were distributed across 144 sources, including journals, books, and conference outlets. The dataset exhibited a strong expansion over time, indicating a rapidly developing research area.

The mean age of documents was 3.23 years, suggesting that the corpus was dominated by recent publications. On average, each document received 16.28 citations, corresponding to 3.37 citations per year per document, reflecting moderate to high scholarly visibility. Collectively, the corpus cited 9,588 references, indicating substantial engagement with prior literature.

In terms of document types, journal articles constituted the majority of the corpus (*n* = 242), followed by book chapters (*n* = 17) and reviews (*n* = 6). Other document types, such as conference papers, editorials, and books, were represented in smaller numbers (see Table 1).

Authorship analysis showed contributions from 592 unique authors, with 732 author appearances across all documents. The mean number of documents per author was 0.50, and the mean number of co-authors per document was 2.50, indicating a predominantly collaborative research structure. While 62 documents were single-authored, the international co-authorship rate of 22.18% pointed to a notable, though not dominant, level of cross-national collaboration.

Taken together, these indicators characterize the corpus as recent, rapidly growing, moderately cited, and collaboration-oriented, providing a robust empirical basis for subsequent thematic and temporal analyses (see Table 2).

**Table 2:** Descriptive summary of the bibliometric corpus (2008–2025).

Indicator	Value
Timespan	2008–2025
Documents	293
Sources (journals, books, etc.)	144
Annual growth rate (%)	25.26
Average document age (years)	3.23
Average citations per document	16.28
Average citations per year per document	3.37
Total references	9,588
Unique authors	592
Author appearances	732
Single-authored documents	62
Documents per author	0.50
Co-authors per document	2.50
International co-authorship	22.18

### 3.1 Growth, productivity patterns, and most influential authors, journals, and country contributions in the field of English language teacher resilience research

**Figure 1:**
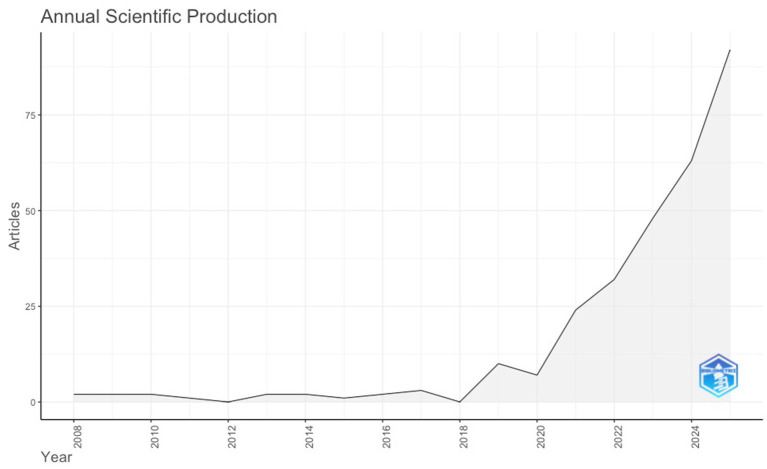
Annual scientific production.

Annual scientific production reveals three discernible phases in the development of English language teacher resilience research. The field remained emergent between 2008 and 2017, with annual output limited to one to three publications, indicating limited research attention and the absence of a consolidated research agenda. A transitional growth phase emerged between 2019 and 2020, during which annual output increased to between 7 and 10 publications, suggesting growing but still moderate engagement with the construct. From 2021 onward, the literature entered a phase of sustained and accelerated expansion. Publication volume rose from 24 documents in 2021 to 32 in 2022, followed by sharp increases to 48 in 2023, 63 in 2024, and 92 in 2025. This period represents the most intensive phase of knowledge production to date and indicates the consolidation of teacher resilience as a central topic within ELT research. The rapid escalation temporally aligns with increased scholarly attention to teacher well-being, burnout prevention, and resilience-building in the post-2020 period, during which the professional sustainability of language teachers became more visible within academic discourse (Figure 1).

**Figure 2:**
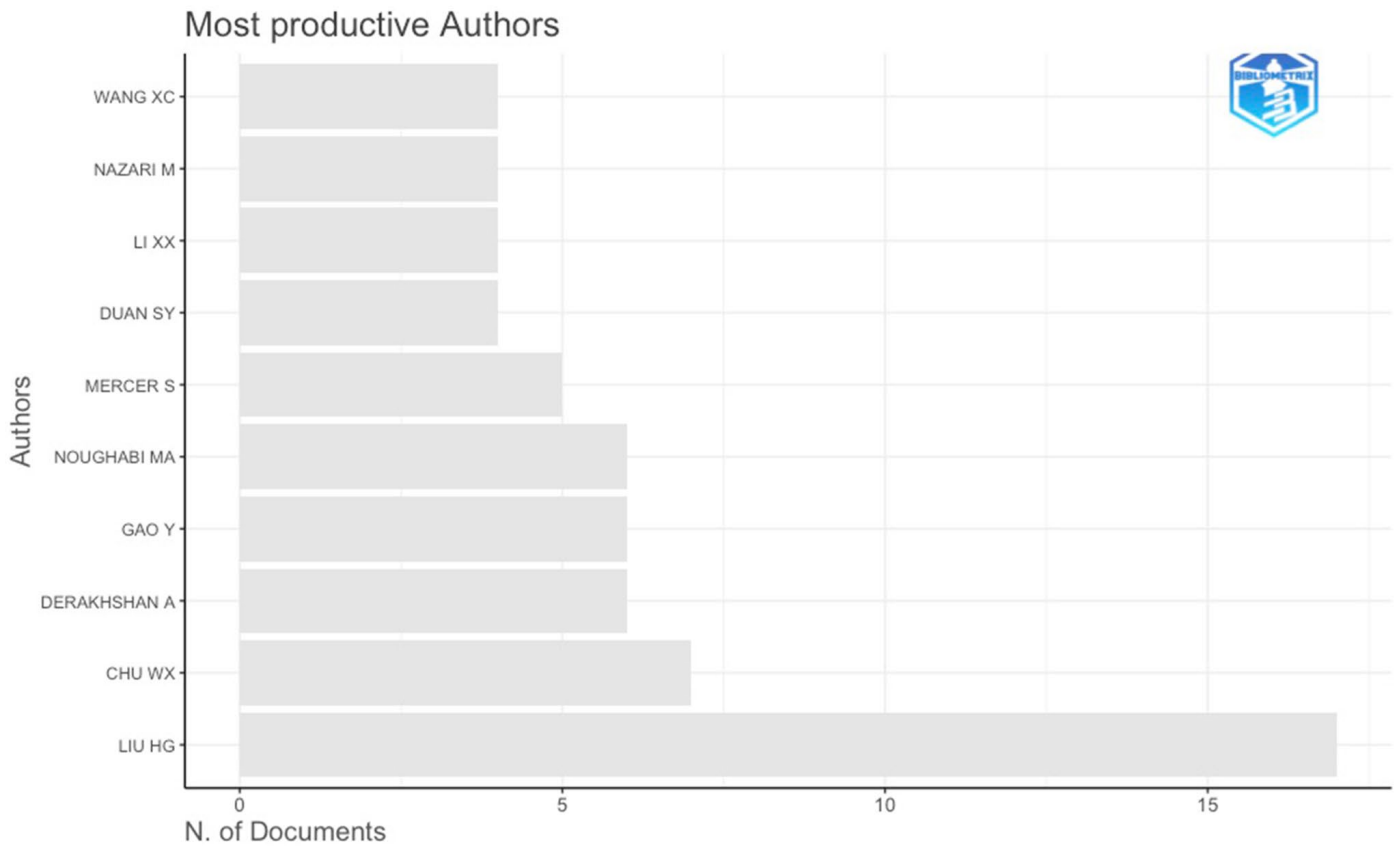
Most productive authors.

Author productivity in the field of English language teacher resilience exhibits a highly uneven distribution, with a small number of scholars contributing multiple publications and a long tail of authors appearing only once. At the top of the distribution, Liu H. G. emerges as the most prolific contributor with 17 publications, followed by Chu W. X. with 7 publications. A second tier of recurring contributors includes Derakhshan A. and Gao Y. (6 publications each), alongside Noughabi M. A. (6 publications) and Mercer S. (5 publications). Beyond this core group, a limited number of authors contributed between three and four publications, while the vast majority of authors in the dataset are represented by a single publication. This distribution indicates a core–periphery authorship structure, in which a small group of researchers has maintained sustained engagement with the topic, whereas most contributors have participated episodically. The prominence of a limited core of repeat authors suggests the emergence of recognized research leaders who have shaped the development of the field over time. At the same time, the large number of single-publication authors reflects the field’s openness to new contributors and its continued expansion across diverse institutional and geographic contexts (Figure 2).

The distribution of publications across journals indicates a moderately concentrated yet institutionally diverse outlet structure for research on English language teacher resilience. A small number of journals function as primary publication venues, while a long tail of sources contributes one or two articles each. At the top of the distribution, Frontiers in Psychology emerges as the most prolific journal with 25 publications, followed closely by the European Journal of Education with 23 publications. A second tier of core journals includes System and the Asian-Pacific Journal of Second and Foreign Language Education (each with 12 publications), both of which are well established within applied linguistics and language education research. Beyond this core group, several journals contribute between five and six publications, including Teaching and Teacher Education, International Journal of Applied Linguistics, Journal of Multilingual and Multicultural Development, and Porta Linguarum. These journals reflect the field’s strong anchoring in teacher education, applied linguistics, and multilingual education, while also indicating engagement with broader educational research communities. At the same time, the presence of a large number of journals represented by only one publication underscores the diffuse and interdisciplinary nature of the field, spanning psychology, education, applied linguistics, sustainability, and social sciences. Overall, the source distribution suggests that English language teacher resilience research is not confined to a single disciplinary journal cluster, but is instead disseminated across a wide range of outlets. This pattern is characteristic of an expanding research area in which conceptual and methodological contributions circulate across multiple academic communities rather than consolidating within a narrowly defined journal niche.

**Figure 3:**
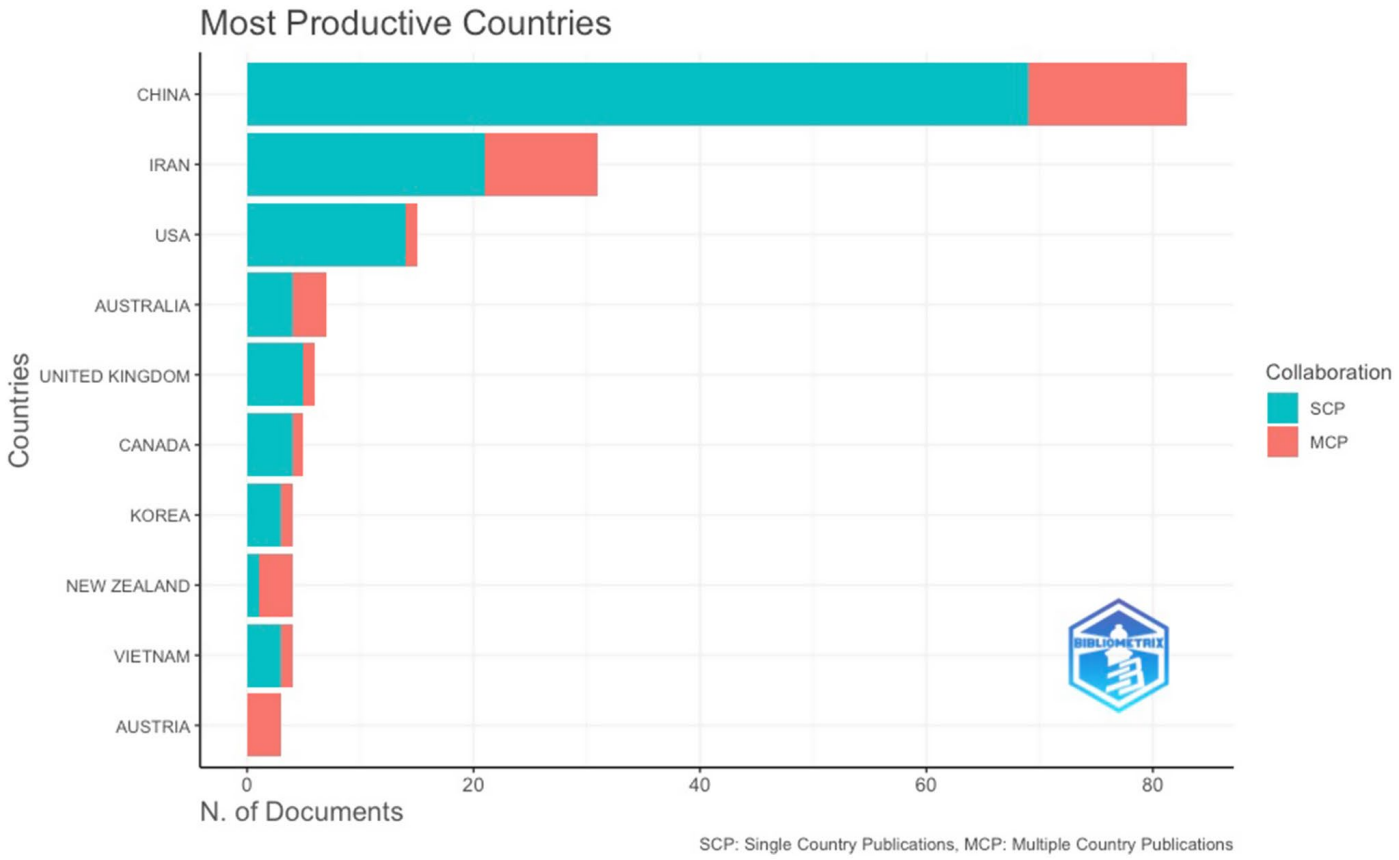
Most productive countries.

Publication patterns were geographically uneven. China accounted for the largest share of publications (83 documents, 28.3% of the 293-document corpus), followed by Iran (31 documents, 10.6%) and the United States (15 documents, 5.1%). A second tier of contributors included Australia (7, 2.4%), the United Kingdom (6, 2.0%), and Canada (5, 1.7%), with Korea, New Zealand, and Vietnam each contributing four documents (1.4% each). Overall, the field showed broad international reach, but knowledge production remained concentrated in a small number of national contexts. International collaboration was generally limited in high-output countries. China produced 69 single-country publications (SCP) and 14 multiple-country publications (MCP), yielding an MCP ratio of 0.17 (14/83). Iran showed comparatively higher collaboration (10 MCP out of 31, MCP ratio = 0.32), whereas the United States exhibited minimal collaboration (1 MCP out of 15, MCP ratio = 0.07). In contrast, several low-output countries displayed high MCP ratios driven by small publication counts. Austria produced 3 documents, all MCP (MCP ratio = 1.00), and New Zealand produced 3 MCP out of 4 documents (MCP ratio = 0.75). Similar small-N effects appeared for Slovakia (2/2 MCP, 1.00) and several single-document countries (e.g., Qatar, Singapore, United Arab Emirates, each 1/1 MCP, 1.00). These patterns indicate that cross-national collaboration existed, but it was not the dominant publication mode in the main producer countries (Figure 3).

Citation impact varied substantially by country. Canada showed the highest mean citation impact (mean TC per article = 103.2), indicating that a small number of Canadian-affiliated publications attracted disproportionate citation attention in the dataset. Iran also demonstrated relatively strong citation performance (38.35 mean citations per article) alongside high output, suggesting both productivity and visibility. Several countries displayed high mean citation values despite low output (e.g., Qatar = 42.0, United Arab Emirates = 25.0, United Kingdom = 25.67), which likely reflected the influence of a small number of highly cited papers rather than sustained high-impact production. Taken together, the country results suggest a concentrated production structure, modest collaboration in the dominant producers, and citation influence that was sensitive to a small number of high-impact publications in lower-output contexts.

### 3.2 Intellectual structure of the field based on author and document co-citation analysis

The most influential works in the dataset collectively foregrounded teacher well-being, emotional experience, positive psychology, and adaptive functioning in language education, with resilience typically treated as an integrative or emergent outcome rather than a standalone construct. Highly cited publications in Frontiers in Psychology and System established a shared emphasis on positive psychological resources that support teachers’ professional functioning. Wang et al. (2021) (Frontiers in Psychology, TC = 559) synthesized positive psychology research in second and foreign language education, highlighting constructs such as enjoyment, emotion regulation, grit, well-being, and resilience as key resources for sustaining engagement and instructional quality. Similarly, Dewaele et al. (2019) (Frontiers in Psychology, TC = 523) examined emotional experiences in language teaching and learning, particularly foreign language enjoyment and classroom anxiety, reinforcing the analytical centrality of teacher and learner emotions.

This orientation was further consolidated by MacIntyre et al. (2020) (System, TC = 500), which documented language teachers’ coping strategies and emotional regulation during the COVID-19–induced shift to online teaching, situating well-being and stress management at the core of teacher psychology research. Subsequent high-impact studies extended this framework by empirically linking positive psychological resources to teaching experiences. Derakhshan et al. (2022) (System, TC = 181) examined resilience, well-being, and L2 grit as predictors of foreign language teaching enjoyment, while Han and Wang (2021) (Frontiers in Psychology, TC = 156) demonstrated the roles of self-efficacy and work engagement in shaping reflective practice. Conceptually adjacent work by Hiver and Dörnyei (2017) (Applied Linguistics, TC = 116) introduced the notion of language teacher immunity, framing adaptive responses to stress as dynamic and potentially stabilizing or constraining professional development.

More recent contributions, such as Wang Y. et al. (2024) (Journal of Multilingual and Multicultural Development, TC = 148), explicitly addressed resilience through a cross-cultural lens, identifying emotional regulation and instructional competence as key challenges in sustaining professional resilience. Overall, the citation structure indicates that the intellectual foundations of English language teacher resilience research are anchored in positive psychology, teacher emotion research, and adaptive views of professional functioning, with the field’s most influential contributions emerging at the intersection of applied linguistics and psychological inquiry.

Author keywords indicated that resilience functioned as the conceptual core of the corpus (56 occurrences). Frequently co-occurring author-supplied terms included EFL teachers (28), positive psychology (22), teacher resilience (18), emotion regulation (14), well-being (13), work engagement (11), and self-efficacy (10), reflecting a strong emphasis on teachers’ emotional, motivational, and psychological resources within language education contexts. Additional keywords such as teacher education, professional development, teacher identity, and mindfulness suggested that resilience research was closely linked to teacher learning, identity formation, and adaptive professional practices. Keywords-Plus analysis reinforced and broadened this thematic structure. Alongside resilience (50 occurrences), high-frequency terms included burnout (22), motivation (15), self-efficacy (14), job satisfaction (12), engagement (11), stress, stressors, and anxiety, indicating sustained attention to work-related pressures and psychological strain. The prominence of students, school, and education further pointed to the institutional embedding of resilience research. Taken together, the overlap between author keywords and Keywords-Plus illustrates an integrated research domain in which resilience is examined at the intersection of emotional and motivational resources and stress- and workload-related constraints, rather than as an isolated psychological attribute.

**Figure 4:**
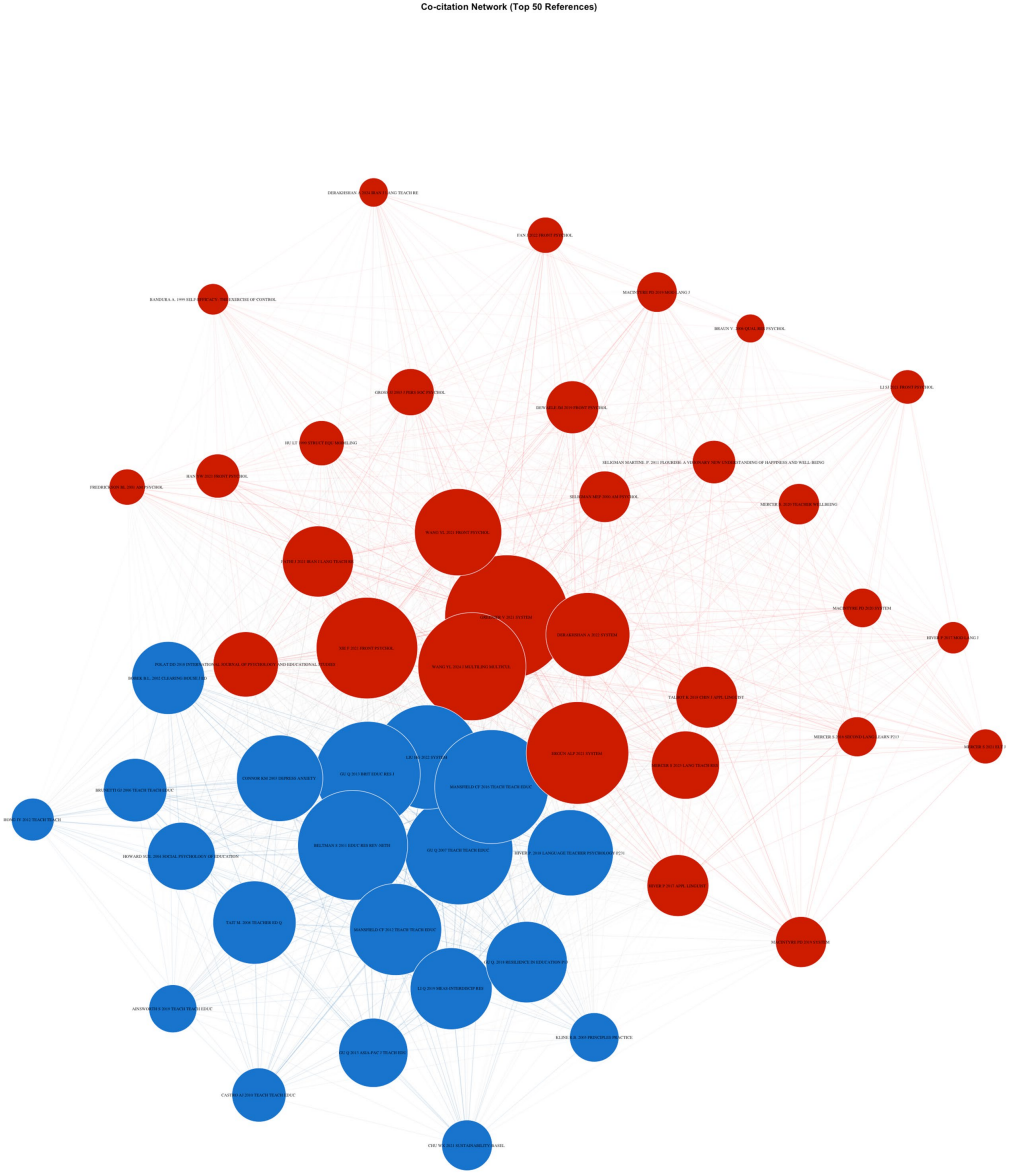
Co-citation network.

Co-citation analysis of the 50 most co-cited references produced a dense network of 1,101 edges with very high density (0.899), indicating a tightly interconnected intellectual structure. Community detection (Louvain) identified two communities with moderate separation (modularity = 0.124), yielding a larger cluster (*n* = 30) and a smaller cluster (*n* = 20). Community detection was performed using the Louvain algorithm as implemented in igraph (v2.2.1) with the default resolution parameter (*γ* = 1.0). A sensitivity analysis across *γ* = 0.50–2.00 indicated that the two-community structure remained stable at *γ* = 0.75–1.00, whereas higher resolution values produced artificial fragmentation. The larger cluster concentrated ELT-specific and applied-linguistics scholarship that integrates resilience with positive psychology and teacher emotion processes, anchored by recent high-centrality works published in System, Frontiers in Psychology, and related journals (e.g., Derakhshan et al., 2022; Ergün and Dewaele, 2021; Greenier et al., 2021; Wang Y. et al., 2024; Wang et al., 2021; Xie, 2021). Within the same community, canonical psychological foundations also appeared among the most central references, including Seligman and Csikszentmihalyi (2000) and Gross and John (2003), indicating that ELT resilience research has been built through explicit uptake of positive psychology and emotion-regulation models rather than solely field-internal theorizing. The second cluster centered on education-based teacher resilience and professional trajectory frameworks, dominated by widely cited foundations in teacher resilience research and measurement traditions (e.g., Beltman et al., 2011; Connor and Davidson, 2003; Gu and Day, 2007; Mansfield et al., 2016), alongside ELT resilience contributions that align closely with these broader education traditions (e.g., Liu and Chu, 2022). Ultimately, the combination of very high density and modest modularity indicates substantial conceptual overlap between ELT-oriented positive-psychology and emotion-focused work and the broader teacher-resilience literature, suggesting that the domain has developed as a largely shared theoretical space rather than as sharply separated subfields (Figure 4).

### 3.3 Research fronts and emerging themes identified through keyword co-occurrence and thematic evolution analysis

**Figure 5:**
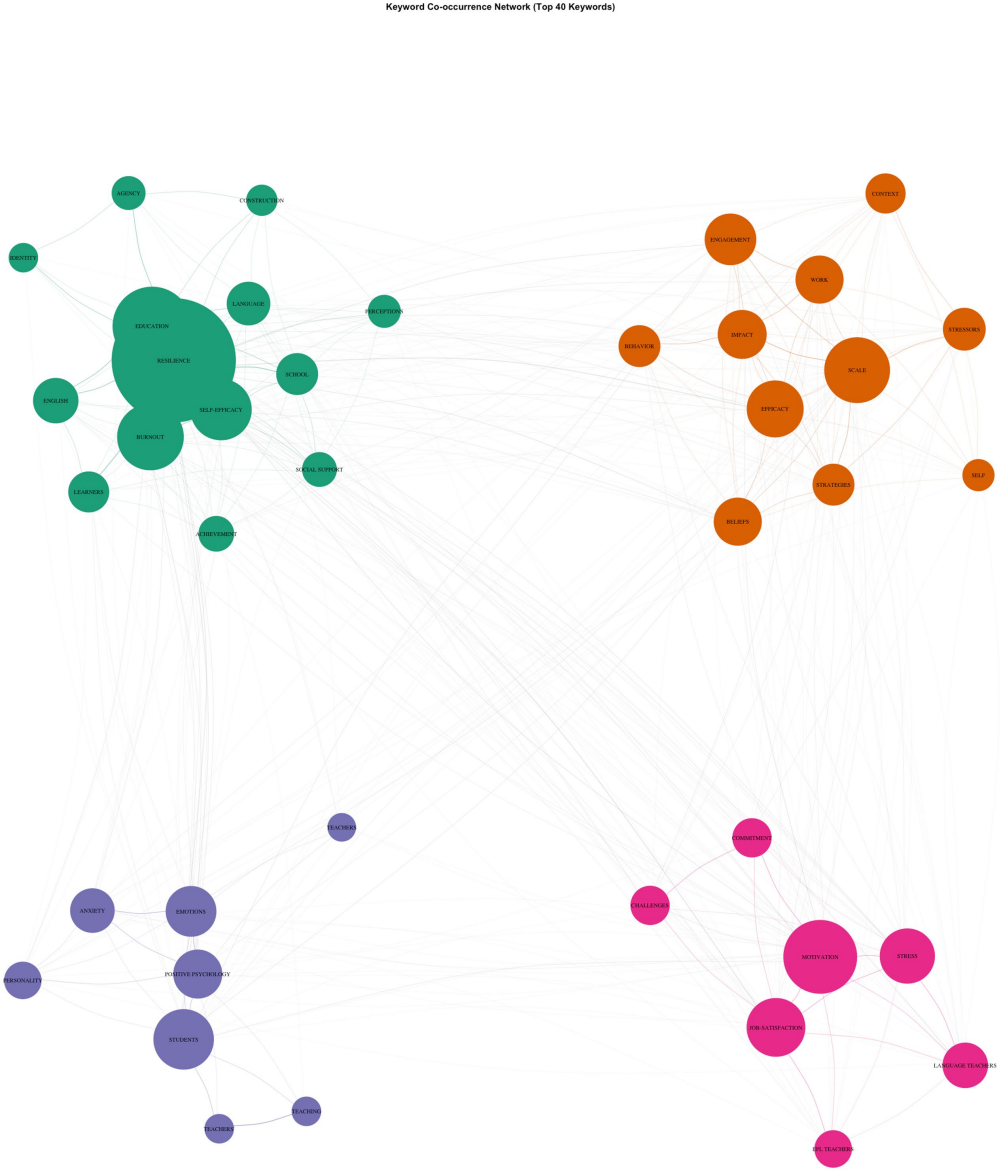
Keyword co-occurrence.

Across the top 40 author keywords, the co-occurrence network was moderately dense (40 nodes, 358 edges, density = 0.459, mean degree = 17.9), indicating that core concepts tended to co-occur widely rather than forming isolated topical silos. Louvain community detection identified four thematic clusters (modularity = 0.153), suggesting meaningful but not sharply segregated thematic organization. Cluster 1 (*n* = 14) represented the conceptual backbone of the corpus. Anchored by resilience (strength = 117), this cluster also included education, burnout, self-efficacy, English, language, school, learners, agency, and social support. Its relatively high internal density (0.59) and the highest total strength (466) indicate that resilience was framed as a central construct situated in educational contexts and closely linked to personal resources, professional functioning, and broad domain descriptors. Cluster 2 (*n* = 11) clustered measurement- and work-resource–oriented constructs, including scale, efficacy, engagement, beliefs, work, stressors, strategies, behavior, context, and impact. This cluster showed high internal cohesion (density = 0.82), reflecting a variable-centered strand of research focused on operationalization, psychological resources, and context-linked explanatory factors, consistent with survey-based and modeling approaches. Cluster 3 (*n* = 8) concentrated on emotional and affective dimensions, bringing together students, emotions, positive psychology, anxiety, learners, personality, and teaching. With moderate internal density (0.61), this cluster highlights research addressing affective experiences and individual differences, situating resilience alongside emotional processes and positive-psychology constructs. Cluster 4 (*n* = 7) reflected a work-psychology and occupational strain orientation, centered on motivation, job satisfaction, stress, language teachers, challenges, commitment, and EFL teachers. Despite its smaller size, this cluster exhibited high internal density (0.81) and high average strength, capturing research that links resilience to job demands, attitudinal outcomes, and professional sustainability in language-teaching contexts. Importantly, the network exhibited substantial cross-cluster connectivity (225 between-cluster edges versus 133 within-cluster edges). Highly influential bridging keywords, such as motivation, resilience, burnout, students, scale, and job satisfaction, showed extensive cross-cluster links, underscoring the integrative nature of the field. Overall, the structure indicates that research on teacher resilience in language education operates as an interconnected thematic space, where educational framing, emotional processes, occupational well-being, and measurement-based approaches are tightly interwoven rather than developing as separate subfields (Figure 5).

**Figure 6:**
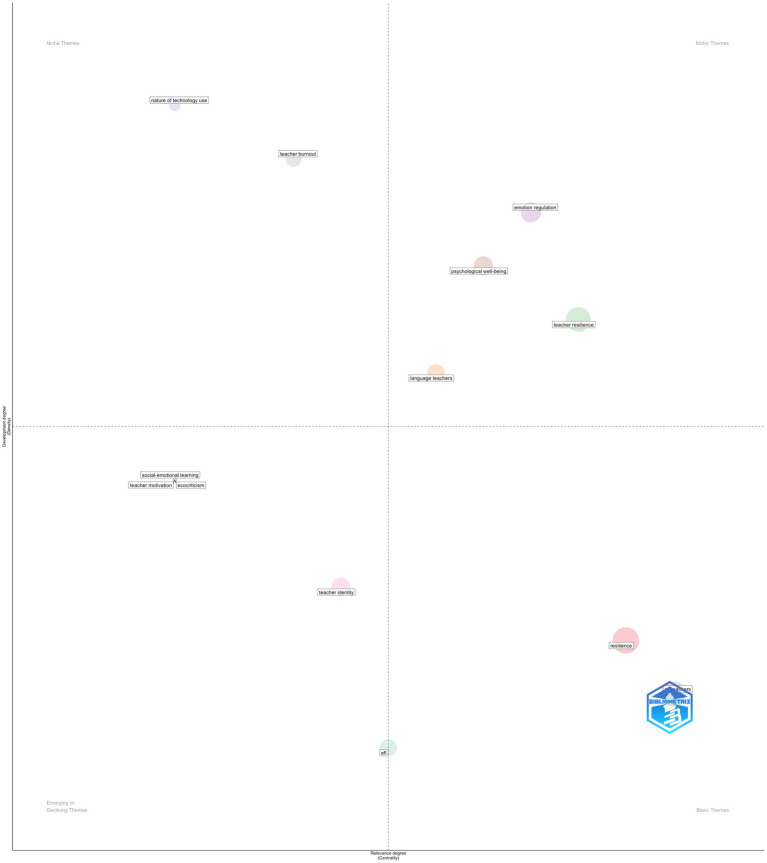
Thematic map.

The thematic map illustrates the conceptual organization of the literature by positioning themes according to their centrality (relevance) and density (development), thereby clarifying both the structural importance and the internal coherence of major research strands. Themes located in the Basic Themes quadrant (high centrality, low-to-moderate density), most notably resilience and EFL teachers, function as the foundational pillars of the corpus. Their high centrality indicates that these concepts are widely connected to other themes across the field, while their comparatively lower density suggests that, although frequently invoked, they serve primarily as broad organizing constructs rather than as tightly elaborated theoretical clusters. This positioning reflects the role of resilience as a pervasive framing concept in language teacher research, often mobilized to contextualize diverse psychological, emotional, and professional variables. The Motor Themes quadrant (high centrality, high density) contains teacher resilience, emotion regulation, psychological well-being, and language teachers. These themes represent the most mature and influential areas of inquiry, combining strong internal development with extensive connections to the wider knowledge structure. Their location indicates that contemporary research has moved beyond treating resilience as a general descriptor and has increasingly focused on its psychological mechanisms, emotional regulation processes, and well-being outcomes within language-teaching contexts. The prominence of these themes suggests that they currently drive theoretical advancement and empirical production in the field. Themes positioned in the Niche Themes quadrant (low centrality, high density), such as teacher burnout and nature of technology use, display strong internal cohesion but limited integration with the broader thematic structure. This pattern implies that these topics are well developed within specialized sub-communities yet remain conceptually peripheral to the core resilience discourse. Their isolation suggests opportunities for stronger theoretical integration, particularly by linking burnout and technology-mediated teaching more explicitly to resilience and well-being frameworks. Finally, the Emerging or Declining Themes quadrant (low centrality, low density) includes teacher motivation, teacher identity, social–emotional learning, ecocriticism, and EFL. These themes appear either to be in early stages of conceptual development or to represent declining lines of inquiry within the current corpus. Their limited centrality indicates weak connectivity to dominant research streams, while low density reflects underdeveloped internal structures. Notably, the presence of teacher motivation and teacher identity in this quadrant suggests that, despite their theoretical relevance, they have not yet been systematically integrated into the resilience-focused research agenda. Further, the presence of “ecocriticism” in the Emerging or Declining quadrant reflects a small cluster of publications linking language teacher resilience to sustainability-oriented and environmental humanities perspectives. Although conceptually peripheral to the core resilience discourse, these studies frame resilience within broader socio-ecological and critical pedagogical contexts. Its low centrality and density indicate that this strand remains marginal and weakly integrated into the dominant intellectual structure of the field. In short, the thematic map portrays a research field that has consolidated around psychologically grounded and well-being–oriented conceptualizations of teacher resilience, while leaving adjacent constructs, such as identity, motivation, and technology use, comparatively under-integrated. This configuration highlights both the maturity of core themes and the potential for future theoretical expansion through greater cross-theme integration (Figure 6).

**Figure 7:**
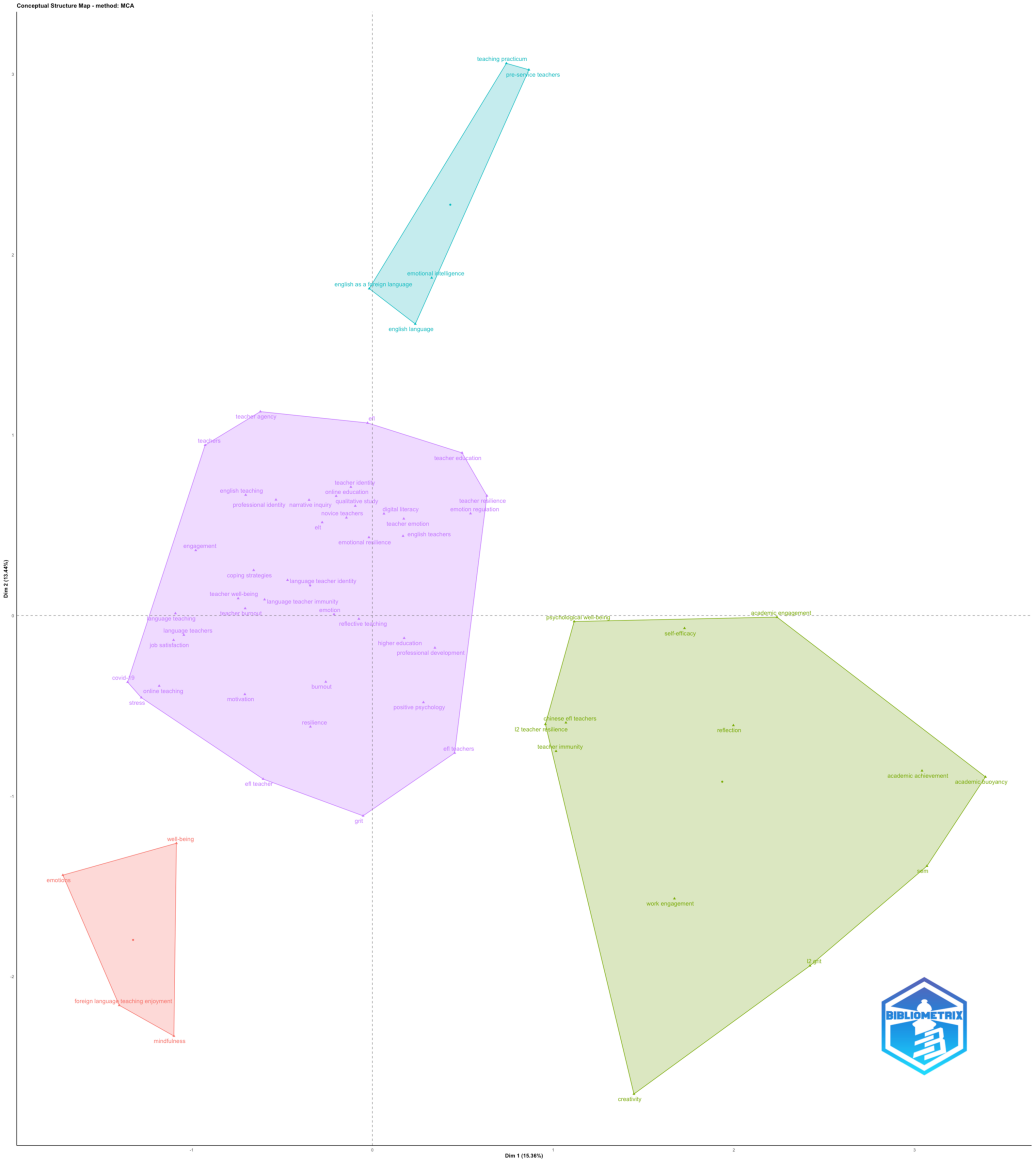
Conceptual structure map.

The conceptual structure map, derived from multiple correspondence analysis, reveals a four-cluster configuration that delineates how keywords co-occur and conceptually organize the literature on teacher resilience and related constructs. The first two dimensions explain a substantive proportion of variance, indicating that the spatial configuration meaningfully represents the conceptual relationships among keywords rather than random dispersion. Cluster 1 constitutes the dominant conceptual nucleus of the map and contains the majority of keywords (*n* = 41). This cluster is anchored by highly contributive and centrally positioned terms such as resilience, stress, teachers, grit, motivation, engagement, burnout, emotion, ELT, and EFL. The concentration of both psychological constructs and contextual identifiers within this cluster suggests that resilience research is primarily framed as a teacher-centered psychological phenomenon embedded in language education contexts. The proximity of burnout, stress, and engagement to resilience indicates that resilience is conceptualized relationally, as a process shaped by emotional demands and professional pressures rather than as a static personal trait. This cluster therefore reflects the field’s core conceptual grammar. Cluster 2 is considerably smaller (*n* = 4) and is defined by emotionally oriented self-regulation constructs, most notably mindfulness and emotions. These keywords display high contributions to the second dimension, positioning the cluster away from the main teacher-resilience axis. This spatial separation indicates that mindfulness-based and emotion-focused approaches form a distinct conceptual subspace, often examined through specialized theoretical lenses rather than integrated into mainstream resilience models. The limited size of the cluster suggests that, while affect regulation is increasingly visible, it remains conceptually segmented within the broader literature. Cluster 3 (*n* = 13) represents a reflective–developmental orientation. Keywords such as reflection, creativity, SEM, and related methodological or developmental constructs characterize this cluster. Its position on the positive side of the first dimension distinguishes it from the stress–burnout–resilience constellation, implying a shift from coping-oriented frameworks toward growth, meaning-making, and methodological elaboration. The presence of reflection and creativity suggests that this cluster captures research examining resilience as a product of reflective practice, cognitive flexibility, and professional learning processes. However, its moderate size and distance from the dominant cluster indicate that these perspectives are complementary rather than central. Cluster 4 (*n* = 5) occupies a peripheral position in the conceptual structure map and contains keywords that are weakly connected to the main conceptual axes. Although smaller and less contributive, this cluster includes terms that point to methodological specificity or emerging conceptual angles, rather than established thematic domains. The spatial marginality of this cluster suggests that these keywords are used in narrower empirical contexts or isolated analytical frameworks, and they do not yet coalesce into a coherent conceptual tradition within the field. In sum, the conceptual structure map indicates that research on teacher resilience is highly centralized around a psychologically grounded, teacher-focused core, with secondary clusters reflecting affect regulation and reflective-developmental approaches. The presence of smaller, peripheral clusters points to conceptual diversification, but the overall structure remains anchored in resilience as a relational construct shaped by stress, motivation, and professional engagement within language education contexts (Figure 7).

**Figure 8:**
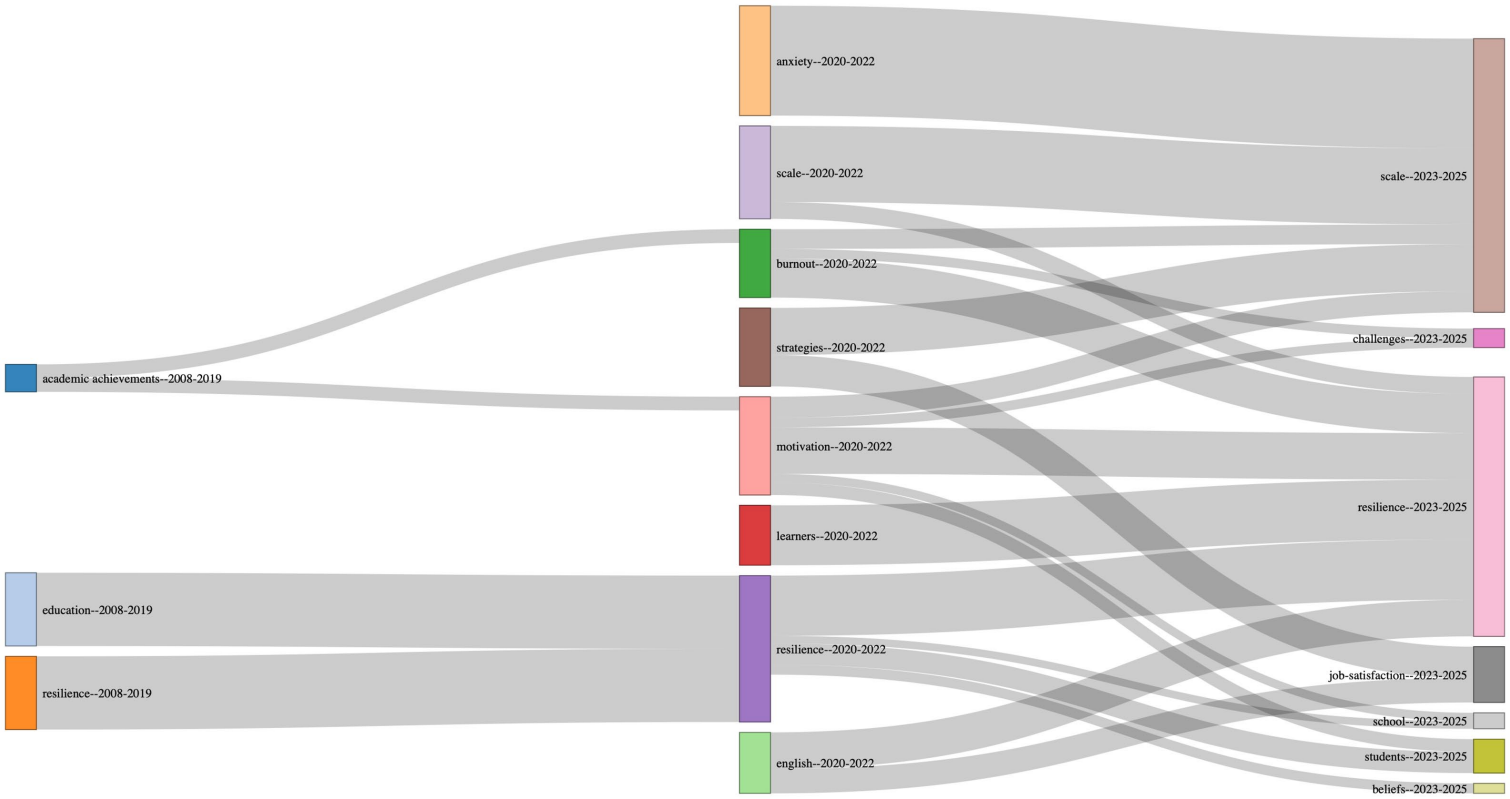
Temporal thematic evolution of the research field across three periods.

To illustrate the longitudinal thematic map and to further answer RQ3, a temporal thematic evolution analysis was conducted using a bibliometric co-word approach across three consecutive periods (2008–2019, 2020–2022, and 2023–2025). The resulting Sankey-style thematic evolution map illustrates both the stability of core themes and the emergence and reconfiguration of thematic emphases over time (Figure 8).

In the initial period (2008–2019), the thematic structure was relatively coarse and weakly differentiated, with broad clusters centered on education, resilience, and academic achievement. These themes exhibited limited forward continuity, indicating an early stage characterized by exploratory and conceptually diffuse research focus.

A marked thematic reorganization occurred during the 2020–2022 period. In this phase, resilience emerged as the dominant and integrative theme, absorbing conceptual continuity from earlier work while simultaneously connecting to a range of psychologically oriented constructs, including burnout, anxiety, motivation, and learners. The appearance and persistence of the scale cluster during this period indicated a shift toward measurement development and construct operationalization, while links to strategies reflected increasing attention to applied and pedagogical responses. Collectively, these patterns signaled a consolidation phase in which resilience functioned as the central organizing construct of the field.

In the most recent period (2023–2025), the thematic structure expanded and differentiated further. Strong continuities from resilience–2020–2022 to resilience–2023–2025 confirmed the sustained centrality of resilience, while additional branches extended toward more specific outcomes and contexts, including job satisfaction, challenges, beliefs, students, and school. This diversification suggested a shift from conceptual and measurement-focused research toward outcome-oriented and context-sensitive inquiries. Notably, the continued prominence of scale across periods pointed to ongoing refinement rather than closure of measurement-related work.

Overall, the Sankey diagram demonstrated a progression from early thematic dispersion to consolidation around resilience, followed by a diversification phase in which resilience served as a stable core supporting multiple specialized research trajectories. This longitudinal pattern provided direct empirical evidence of thematic evolution and complemented the static thematic map (see Figure 6) and keyword co-occurrence (see Figure 5) by revealing how dominant themes persisted, integrated, and expanded over time.

## 4 Discussion

This study provided a multi-database bibliometric mapping of research on English language teacher resilience to examine the field’s growth, intellectual foundations, and thematic organization. By integrating records from WoS and Scopus, the analysis offered a validated overview of how research on ELT teacher resilience has evolved, how it has structured its core knowledge base, and which conceptual directions currently dominate the research landscape. The results indicate that the field has entered a phase of rapid expansion and thematic consolidation, while also revealing persistent structural and conceptual imbalances that constrain cumulative knowledge building.

The high degree of convergence observed across Web of Science and Scopus should not be interpreted as negating the value of multi-database analysis. Rather, it confirms that major developmental signals in ELT resilience research are robust, while simultaneously demonstrating that single-database mappings are insufficient for identifying what the field bibliometrically neglects. The merged corpus reveals that constructs such as teacher identity, motivation, and technology use occupy peripheral or emerging positions despite their theoretical centrality in language teacher psychology. These patterns were attenuated or unstable in smaller or single-source datasets, underscoring that convergence validates integration rather than obviating it.

### 4.1 Growth and consolidation in the field

The annual production curve of the multi-database corpus indicates a late but rapid consolidation of English language teacher resilience research. This trajectory is consistent with large-scale bibliometric research reporting differentiated growth across subfields (Li and Chen, 2024), but the steeper post-2021 increase suggests that ELT resilience has developed as a downstream specialization that consolidated once resilience became more central to discussions of teacher well-being and sustainability (Gu and Day, 2007; Li and Chen, 2024). In contrast to Ekizer’s (2024) ELT-focused mapping, which portrayed the L2-specific evidence base as limited, with only 24 articles retained after screening, the present corpus yields substantially greater volume, indicating that database scope and retrieval strategy materially shape judgments of field maturity and function as a form of bibliometric validation through convergent coverage.

Author productivity further demonstrates a concentrated leadership structure, with a small core of prolific contributors and a long tail of one-time authors. A limited number of researchers account for a disproportionate share, consistent with prior bibliometric evidence that resilience research develops around clustered research groups rather than evenly distributed authorship (Li and Chen, 2024). Substantively, this configuration suggests that conceptual development has been shaped by a stable core, while continued entry from adjacent communities reflects the construct’s interdisciplinary positioning between applied linguistics, teacher education, and psychology.

The journal structure further supports this hybrid disciplinary identity. Psychology-oriented and applied linguistic journals jointly constitute the core publication venues, extending earlier observations that ELT resilience research aligns resilience with well-being, stress, coping, and engagement (Ekizer, 2024). The stronger representation of psychology journals in the present dataset suggests that ELT resilience increasingly functions as an interface topic, where language-teaching questions are framed through psychological constructs rather than remaining confined to applied linguistics. This diffusion across journals strengthens the methodological rationale for multi-database mapping, as single-index coverage would likely underestimate both volume and structure.

Country-level productivity shows pronounced concentration alongside modest international collaboration. China is the dominant producer, followed by Iran and the United States, with collaboration indicators indicating largely nationally anchored research agenda. Considering China and Iran are EFL-dominant contexts where English functions as a foreign rather than a native language, several structural and sociocultural factors may account for this pattern. First, English language education occupies a high-stakes position in both systems, closely tied to university entrance examinations, international mobility, and socioeconomic advancement. Such high institutional pressure plausibly amplifies teachers’ exposure to workload intensity, accountability demands, and performance scrutiny, conditions that align directly with resilience-related constructs such as coping, emotional regulation, and professional sustainability. Second, both countries have experienced rapid expansion in higher education and research output over the past two decades, accompanied by strong incentives for international publication. This expansion has likely accelerated empirical production in applied linguistics and teacher psychology, including resilience research. At the sociocultural level, EFL teachers in China and Iran often operate within centralized curricular frameworks and examination-driven environments, which may heighten tensions between pedagogical autonomy and systemic constraints. Resilience, therefore, may emerge as a particularly salient construct for understanding teacher functioning in these contexts. In addition, positive psychology and teacher emotion research have gained substantial visibility in Asian and Middle Eastern applied linguistics scholarship, providing a theoretical infrastructure that supports resilience-oriented inquiry. However, the geographic concentration of publications raises important epistemological considerations. If empirical evidence is disproportionately generated within specific sociocultural systems, prevailing conceptualizations of resilience may reflect context-bound stressors, institutional norms, and coping repertoires. Constructs operationalized in examination-oriented or centralized educational systems may not fully capture resilience processes in decentralized, multilingual, or resource-diverse contexts. Consequently, while the productivity of China and Iran has significantly advanced the field, broader cross-cultural validation remains necessary to ensure conceptual generalizability and theoretical refinement. Further, while some contexts exhibit higher international co-authorship, overall MCP ratios remain low, supporting earlier claims that growth in resilience research does not automatically translate into stable multinational collaboration networks (Li and Chen, 2024).

Citation impact patterns further indicate that influence is unevenly distributed, with low-output countries achieving disproportionately high visibility. This asymmetry mirrors prior results in teacher resilience research and suggests that a small number of highly cited contributions exert outsized influence on conceptual and methodological norms (Li and Chen, 2024). Given ongoing concerns about construct heterogeneity and operational variation (Daniilidou and Pezirkianidis, 2025; Zhang, 2023) such concentration may both stabilize and constrain the field’s interpretive range.

Taken together, these results indicate that English language teacher resilience has entered a high-growth but structurally uneven phase, characterized by rapid expansion, concentrated authorship, limited collaboration, and cross-disciplinary diffusion. Compared with earlier ELT-focused mapping that characterized the evidence base as thin (Ekizer, 2024), the present multi-database corpus depicts a substantially larger and more consolidated literature, supporting the use of convergent database coverage as a necessary step for robust bibliometric validation.

### 4.2 Intellectual foundations of the field: positive psychology, teacher emotion, and adaptive functioning

The co-citation structure reveals a highly integrated intellectual foundation for English language teacher resilience research, characterized by extensive cross-referencing among influential works rather than fragmentation into competing theoretical camps. High network density and moderate modularity indicate that core publications are extensively co-cited and that conceptual boundaries between clusters remain permeable. This pattern suggests cumulative knowledge building grounded in a shared body of literature rather than parallel or competing traditions.

At the level of highly cited documents, the intellectual center of the field is anchored in positive psychology, teacher emotion research, and adaptive models of professional functioning. Influential studies foreground emotional regulation, enjoyment, well-being, and coping as central explanatory resources in language teaching contexts, positioning resilience as an emergent and relational outcome rather than an isolated construct. Subsequent empirical studies (e.g., Derakhshan et al., 2022; Han and Wang, 2021) reinforce this framing by linking resilience to self-efficacy, engagement, reflective practice, and teaching enjoyment, indicating broad consensus around an integrative conceptualization consistent with dynamic and developmental perspectives. Although one author (Liu H. G.) contributed 5.8% of the corpus (17/293 documents), a sensitivity analysis excluding these publications yielded substantively identical thematic rankings and a comparable co-citation modularity from 0.124 to 0.115. This indicates that the intellectual structure of the field is not reducible to single-author dominance.

The co-citation communities further clarify how this integration is organized. The larger community concentrates ELT-specific research that explicitly incorporates positive psychology and teacher emotion frameworks, drawing on both applied linguistics psychology journals. The co-presence of canonical psychological references (e.g., Gross and John, 2003; Seligman and Csikszentmihalyi, 2000) alongside ELT-focused studies indicates substantive theoretical integration, not merely terminology borrowing. A second co-citation community centers on broader education-based resilience traditions, including foundational work by Gu and Day (2007, 2013), Beltman et al. (2011), Mansfield et al. (2016), and Connor and Davidson (2003). These studies emphasize resilience as a multidimensional, socioecological construct shaped by professional trajectories, institutional conditions, and personal resources. The co-presence of ELT-specific contributions (e.g., Liu and Chu, 2022) within this community suggests continued theoretical anchoring in general teacher resilience frameworks.

Importantly, the relatively low modularity between these two communities indicates substantial conceptual overlap rather than theoretical separation, extending earlier bibliometric results that describe teacher resilience as a shared, cross-disciplinary construct (Li and Chen, 2024). It also extends Ekizer’s (2024) ELT-focused mapping by showing that the apparent thematic diversity of the literature is underpinned by a tightly connected citation base, suggesting conceptual coherence beneath surface-level topical variation.

Keyword analyses corroborate this interpretation. The recurrent co-occurrence of resilience with emotion regulation, well-being, work engagement, self-efficacy, burnout, and motivation situates the construct at the intersection of psychological resources and work-related stressors. The convergence between author keywords and Keywords-Plus further suggests that this integrative framing is stable across journals and databases rather than author-specific. At the same time, this overlap supports ongoing concerns about conceptual diffusion as resilience is frequently operationalized alongside closely related constructs without explicit theoretical boundaries (Daniilidou and Pezirkianidis, 2025; Zhang, 2023).

Overall, the co-citation structure indicates that English language teacher resilience research has evolved as a theoretically cohesive and integrative domain rooted in positive psychology and teacher emotion research, while remaining firmly connected to broader education-based resilience frameworks. This structural cohesion supports cumulative theory development, but it also underscores the need for clearer conceptual boundaries to prevent construct drift as the literature continues to expand.

### 4.3 Integrated research fronts and thematic organization of teacher resilience in ELT

The keyword co-occurrence and thematic analyses indicate that English language teacher resilience research has developed as a densely interconnected and integrative thematic domain, rather than a collection of isolated topical strands. Network density and degree statistics show that core concepts repeatedly co-appear, while relatively low modularity indicates thematic boundaries. Taken together, these properties suggest a field organized around shared explanatory concerns in which psychological, emotional, and contextual constructs are routinely examined in combination. Longitudinal thematic evolution further indicates that this interconnectedness reflects progressive thematic convergence rather than static overlap.

At the cluster level, the thematic structure is differentiated yet interdependent. The largest and most influential cluster centers on resilience as the field’s conceptual backbone, integrating educational contexts, psychological resources, and occupational strain. This configuration confirms that resilience in ELT is framed as a relational and context-embedded construct, consistent with socioecological framings emphasizing interactions between personal capacities and contextual affordances (Beltman et al., 2011; Gu and Day, 2007). The longitudinal evolution map corroborates this interpretation by showing that resilience absorbed continuity from earlier, more diffuse themes and remained the dominant organizing construct across all periods.

A second cluster reflects a tightly integrated measurement- and resource-oriented research front, characterized by intensive co-use of scales and related psychological constructs. Its cohesion aligns with earlier observations that ELT resilience research relies heavily on self-report instruments and cross-sectional designs (Daniilidou and Pezirkianidis, 2025; Ekizer, 2024). Longitudinally, the sustained prominence of measurement-related themes indicates ongoing refinement rather than thematic closure, while strong interconnections with other clusters suggest that measurement practices serve to operationalize constructs drawn from emotional, motivational, and occupational frameworks rather than forming an isolated methodological subdomain.

Affect-oriented constructs form a third cluster that remains closely coupled with resilience core. This configuration reflects the field’s sustained engagement with emotions, well-being, and anxiety as explanatory mechanisms, echoing patterns identified in the co-citation analyses that foreground emotion regulation and psychological well-being (Dewaele et al., 2019; Wang et al., 2021). Longitudinal evidence indicates that affective constructs became more tightly integrated with resilience during the consolidation phase, reinforcing the field’s shift toward psychologically grounded explanations.

A smaller but internally cohesive cluster links resilience to distinct occupational outcomes, including motivation, job satisfaction, challenges, and professional identity. Although secondary in size, this strand highlights an outcome-oriented framing in which resilience is connected to sustainability and retention rather than emotional coping alone (Gu and Day, 2013; Mansfield et al., 2016). Its increased visibility in the most recent period reflects a diversification phase in which resilience research extends toward professional outcomes and institutional contexts without displacing the core psychological framing.

Across clusters, the prevalence of between-cluster connections and the bridging role of key constructs (e.g., resilience, motivation, burnout, scale, job satisfaction) indicate that research fronts are structurally integrated rather than isolated. Longitudinal flow patterns reinforce this interpretation by showing that dominant constructs persist across periods while being recontextualized within evolving thematic configurations.

The thematic map further clarifies the developmental status of these research fronts. Basic themes such as resilience displayed high centrality but lower density, indicating their role as foundational framing concepts rather than internally elaborated theoretical constructs. In contrast, motor themes such as teacher resilience, emotion regulation, and psychological well-being combined high centrality with high density, marking them as the most mature and theoretically productive areas of inquiry. This pattern aligns with temporal evidence of early dispersion, mid-phase consolidation, and recent diversification anchored by a stable resilience core.

At the same time, constructs such as teacher motivation and identity remain weakly integrated despite their theoretical relevance, while niche themes such as burnout and technology show strong internal development but limited connectivity. Longitudinal evidence suggests that these themes are intermittently absorbed into broader resilience-centered trajectories, pointing to uneven theoretical integration rather than decline.

The placement of teacher identity and teacher motivation in the emerging or declining quadrant requires careful methodological interpretation. In thematic mapping, low centrality and density do not indicate theoretical insignificance but rather limited co-occurrence connectivity within the specific bibliometric network constructed from the selected corpus. In broader applied linguistics scholarship, teacher identity constitutes a well-established and theoretically central construct, frequently linked to agency, professional development, and emotional labor. Its peripheral position in the present map therefore likely reflects network dispersion rather than conceptual decline.

Two explanations are plausible. First, identity-related work may be lexically distributed across adjacent constructs such as agency, beliefs, professional development, or emotion regulation. If identity is operationalized indirectly through these constructs, its standalone keyword frequency and co-occurrence density will be attenuated. Second, the temporal thematic evolution supports partial thematic absorption rather than decline. In the mid-period (2020–2022), resilience consolidates as a dominant hub, while related constructs such as motivation and strategies feed into the resilience trajectory in the 2023–2025 phase. Identity appears less as an independent cluster and more as a relational construct embedded within resilience-centered inquiry. This suggests integration into broader resilience frameworks rather than thematic disappearance.

A similar interpretation applies to teacher motivation. Although positioned peripherally in the static map, temporal flows indicate that motivation contributes to resilience-centered development in later periods. The apparent weak integration therefore reflects structural network positioning within this specific corpus, not reduced scholarly relevance.

Accordingly, the lower-left quadrant should be interpreted as indicating incomplete bibliometric consolidation rather than theoretical marginality. These results point to uneven keyword standardization and conceptual overlap across resilience-related research streams, underscoring the importance of cautious interpretation of automated thematic classification.

Taken together, the static and longitudinal analyses provide converging evidence that ELT resilience research has progressed from exploratory dispersion to theoretical consolidation and, more recently, to outcome- and context-sensitive diversification. The field has consolidated around a psychologically grounded, relational understanding of resilience, while leaving adjacent constructs unevenly connected, highlighting clear directions for future integrative work.

## 5 Conclusion, limitations, and implications for future ELT resilience synthesis and empirical research

The present study provided a multi-database bibliometric consolidation of English language teacher resilience research, offering field-level validation of its growth trajectory, intellectual foundations, and thematic organization. The results demonstrated that ELT resilience research has transitioned from an emergent topic into a rapidly expanding and increasingly visible research domain, particularly since 2021. This expansion coincided with intensified scholarly attention to teacher well-being, emotional labor, and professional sustainability, reflecting broader educational and societal concerns amplified in the post–COVID-19 period.

Across bibliometric indicators, the field exhibited a clear core–periphery structure. A small group of highly productive authors, journals, and countries accounted for a disproportionate share of output and influence, while a large number of contributors participated episodically. Co-citation analysis further revealed that the intellectual foundations of ELT resilience research are not anchored in resilience as an isolated construct, but rather in an integrated theoretical space shaped by positive psychology, teacher emotion research, and adaptive views of professional functioning. Canonical psychological frameworks were actively appropriated alongside applied linguistics scholarship, indicating sustained cross-disciplinary knowledge transfer.

At the thematic level, keyword co-occurrence, thematic mapping, and conceptual structure analyses consistently showed that resilience operates as a relational and integrative construct, embedded within emotional regulation, occupational strain, motivational resources, and measurement-oriented research traditions. Rather than fragmenting into discrete subfields, the literature formed a densely connected thematic space in which resilience, well-being, stress, engagement, and professional resources were tightly interwoven. Taken together, these results position ELT resilience research as a conceptually cohesive yet methodologically diverse domain, characterized by rapid growth, strong psychological grounding, and expanding empirical scope.

Several limitations should be acknowledged when interpreting the results. First, although the study employed a multi-database bibliometric approach to address concerns regarding single-source dependency, the analysis remained constrained by the coverage, indexing practices, and metadata quality of the selected databases. Relevant publications indexed outside these databases, including regional journals or non-English journals, may therefore be underrepresented.

Second, bibliometric techniques capture structural and relational patterns in the literature rather than the substantive quality or theoretical depth of individual studies. High citation counts and centrality indicators reflect visibility and influence, but they do not necessarily equate to conceptual rigor or methodological robustness. As such, the present results should be interpreted as indicators of field-level organization rather than evaluative judgments of research quality.

Third, keyword-based analyses relied on author-supplied terms and Keywords Plus, which are subject to inconsistencies in labeling, synonym use, and conceptual granularity. Although normalization procedures were applied, some degree of semantic overlap and construct ambiguity is inherent in large-scale bibliometric mapping. Further, the study focused on published literature and did not incorporate dissertations, or policy documents, which may play an important role in shaping applied understandings of teacher resilience in practice-oriented contexts.

Finally, a methodological limitation of the present study concerns the timing of database queries. The Web of Science search was conducted on December 4, 2025, whereas the Scopus search was conducted approximately 6 weeks later, on January 17, 2026. Although a year filter excluding 2026 publications was applied to the Scopus search to preserve temporal alignment, the two databases were not queried at the exact same time point. As bibliographic databases are continuously updated, this temporal offset may have introduced minor asymmetries in record availability or metadata indexing. However, given the high convergence observed across temporal trends, thematic structures, and geographic distributions, this discrepancy is unlikely to have materially affected the main bibliometric patterns reported in the study.

The results of this study carry several implications for advancing ELT resilience research at both the synthesis and empirical levels.

Future bibliometric and review-based syntheses would benefit from greater conceptual standardization. The persistent overlap between resilience, protective factors, outcomes, and adjacent constructs underscores the need for shared coding frameworks that explicitly distinguish resilience from its antecedents and correlates. Applying reusable typologies, such as trait-, process-, and context-oriented perspectives, would enhance cross-review comparability and reduce construct drift.

Moreover, the dense thematic connectivity observed in this study suggests that future syntheses should move beyond cataloging determinants toward mapping relational configurations among emotional, motivational, and occupational variables. Longitudinal bibliometric techniques, thematic evolution analysis, and multi-database validation should be prioritized to capture how resilience research continues to reorganize over time.

At the empirical level, the predominance of cross-sectional designs identified in the literature points to an urgent need for longitudinal, developmental, and mixed-methods approaches. Given that resilience is consistently framed as dynamic and process-oriented, future studies should align their designs with this conceptualization by examining change, accumulation, and fluctuation across career stages and instructional contexts.

Additionally, the thematic marginality of constructs such as teacher identity, motivation, and technology-mediated teaching suggests underexplored opportunities for theoretical integration. Empirical research that explicitly models how these constructs interact with emotional regulation, occupational stress, and institutional conditions could substantially deepen explanatory power. Greater emphasis on cross-national collaboration and comparative designs would help address the geographic concentration identified in the field and support the development of more generalizable resilience frameworks.

Finally, the results also carry concrete implications for teacher education programs, schools, and educational authorities. First, given the central role of emotion regulation, psychological well-being, and occupational stress in the resilience network, teacher education curricula should incorporate structured training in emotional competence, stress management, and reflective practice rather than treating resilience as an implicit by-product of experience. Pre- and in-service programs could embed resilience-oriented modules that integrate case-based reflection, mentoring systems, and guided peer collaboration to strengthen adaptive coping repertoires. At the institutional level, schools should move beyond individual-level interventions and address structural stressors by ensuring manageable workloads, transparent evaluation systems, and access to professional support mechanisms such as supervision or counseling. Educational authorities, in turn, should formalize resilience as part of professional standards and continuous professional development frameworks, aligning policy with evidence that resilience is context-sensitive and environmentally shaped. Investments in collaborative school cultures, mentoring structures for early-career teachers, and psychologically informed leadership training may therefore yield more sustainable effects than isolated well-being workshops. Such multi-level action, spanning curriculum, school climate, and policy design, would translate resilience research into systemic professional support rather than individual responsibility.

## Data Availability

The original contributions presented in the study are included in the article/supplementary material, further inquiries can be directed to the corresponding author.

## References

[ref1] Aria M. Cuccurullo C. (2017). Bibliometrix: an R-tool for comprehensive science mapping analysis. J. Informetr. 11, 959–975. doi: 10.1016/j.joi.2017.08.007

[ref2] Ayoobiyan H. Rashidi N. (2021). Can reflective teaching promote resilience among Iranian EFL teachers? A mixed-method design. Reflect. Pract. 22, 293–305. doi: 10.1080/14623943.2021.1873758

[ref3] Bai J. Wan Z. Wu Z. Peng Q. (2025). Global research trends in metabolism-related intraocular malignancies: a multi-database bibliometric analysis and cross-validation study. Front. Mol. Biosci. 12:1683864. doi: 10.3389/fmolb.2025.1683864, 40994549 PMC12454098

[ref4] Beltman S. Mansfield C. Price A. (2011). Thriving not just surviving: a review of research on teacher resilience. Educ. Res. Rev. 6, 185–207. doi: 10.1016/j.edurev.2011.09.001

[ref5] Chansanam W. Li C. (2025). KKU-BiblioMerge: a novel tool for multi-database integration in bibliometric analysis. Iberoam. J. Sci. Meas. Commun. 5, 1–16. doi: 10.47909/ijsmc.157

[ref6] Connor K. M. Davidson J. R. T. (2003). Development of a new resilience scale: the Connor-Davidson resilience scale (CD-RISC). Depress. Anxiety 18, 76–82. doi: 10.1002/da.10113 12964174

[ref7] Daniilidou A. Pezirkianidis C. (2025). A scoping review of psychometric instruments measuring teachers’ resilience. Encyclopedia 5:109. doi: 10.3390/encyclopedia5030109

[ref8] De Battisti F. Salini S. (2013). Robust analysis of bibliometric data. Stat. Methods Appl. 22, 269–283. doi: 10.1007/s10260-012-0217-0

[ref9] Derakhshan A. Dewaele J.-M. Azari Noughabi M. (2022). Modeling the contribution of resilience, well-being, and L2 grit to foreign language teaching enjoyment among Iranian English language teachers. System 109:102890. doi: 10.1016/j.system.2022.102890

[ref10] Dewaele J.-M. Chen X. Padilla A. M. Lake J. (2019). The flowering of positive psychology in foreign language teaching and acquisition research. Front. Psychol. 10:2128. doi: 10.3389/fpsyg.2019.02128, 31607981 PMC6769100

[ref11] Donthu N. Kumar S. Mukherjee D. Pandey N. Lim W. M. (2021). How to conduct a bibliometric analysis: an overview and guidelines. J. Bus. Res. 133, 285–296. doi: 10.1016/j.jbusres.2021.04.070

[ref12] Ekizer F. N. (2024). Resilience Among L2 Teachers: Mapping Research Trends Through a Biblio-Systematic Review. *SSRN*. doi: 10.2139/ssrn.5059542

[ref13] Entesari E. Yousefi M. H. Eslami H. (2020). A mixed-method study of Iranian EFL teachers’ achieving resiliency: implications for teacher development. Asian-Pac. J. Second. Foreign. Lang. Educ. 5. doi: 10.1186/s40862-020-00096-w

[ref14] Ergün A. L. P. Dewaele J.-M. (2021). Do well-being and resilience predict the foreign language teaching enjoyment of teachers of Italian? System 99:102506. doi: 10.1016/j.system.2021.102506

[ref15] Fredrickson B. L. (2001). The role of positive emotions in positive psychology: the broaden-and-build theory of positive emotions. Am. Psychol. 56, 218–226. doi: 10.1037//0003-066x.56.3.218, 11315248 PMC3122271

[ref16] Greenier V. Derakhshan A. Fathi J. (2021). Emotion regulation and psychological well-being in teacher work engagement: a case of British and Iranian English language teachers. System 97:102446. doi: 10.1016/j.system.2020.102446

[ref17] Gross J. J. John O. P. (2003). Individual differences in two emotion regulation processes: implications for affect, relationships, and well-being. J. Pers. Soc. Psychol. 85, 348–362. doi: 10.1037/0022-3514.85.2.348, 12916575

[ref18] Gu Q. (2018). “(Re)conceptualising teacher resilience: a social-ecological approach to understanding teachers’ professional worlds,” in Resilience in Education: Concepts, Contexts and Connections, eds. Wosnitza M. Peixoto F. Beltman S. Mansfield C. F. (Cham: Springer International Publishing), 13–34.

[ref19] Gu Q. Day C. (2007). Teachers resilience: a necessary condition for effectiveness. Teach. Teach. Educ. 23, 1302–1316. doi: 10.1016/j.tate.2006.06.006

[ref20] Gu Q. Day C. (2013). Challenges to teacher resilience: conditions count. Br. Educ. Res. J. 39, 22–44. doi: 10.1080/01411926.2011.623152

[ref21] Han Y. Wang Y. (2021). Investigating the correlation among Chinese EFL teachers’ self-efficacy, work engagement, and reflection. Front. Psychol. 12:763234. doi: 10.3389/fpsyg.2021.763234, 34803845 PMC8603392

[ref22] Heng Q. Chu L. (2023). Self-efficacy, reflection, and resilience as predictors of work engagement among English teachers. Front. Psychol. 14:1160681. doi: 10.3389/fpsyg.2023.1160681, 37251052 PMC10213630

[ref23] Hiver P. Dörnyei Z. (2017). Language teacher immunity: a double-edged sword. Appl. Linguist. 38, 405–423. doi: 10.1093/applin/amv034

[ref24] Kim Y. Kim T.-Y. (2024). The interplay of teacher resilience and professional development: the case of two beginning EFL teachers in South Korea. Porta Linguarum IX, 173–192. doi: 10.30827/portalin.viix.29889

[ref25] Li X. Chen J. (2024). Survive and thrive in the time of changes: a bibliometric review of teacher resilience, 1998–2023. Rev. Educ. Res. 96, 176–214. doi: 10.3102/00346543241293786

[ref26] Liu H. (2024). Uncovering the mediating role of resilience between EFL teacher turnover intention and wellbeing: a conservation-of-resources theory perspective. System 124:103394. doi: 10.1016/j.system.2024.103394

[ref27] Liu H. Chu W. (2022). Exploring EFL teacher resilience in the Chinese context. System 105:102752. doi: 10.1016/j.system.2022.102752

[ref28] Liu H. Chu W. Duan S. Li X. (2024). Measuring language teacher resilience: scale development and validation. Int. J. Appl. Linguist. 34, 1283–1299. doi: 10.1111/ijal.12562

[ref29] MacIntyre P. D. Gregersen T. Mercer S. (2020). Language teachers’ coping strategies during the COVID-19 conversion to online teaching: correlations with stress, wellbeing and negative emotions. System 94:102352. doi: 10.1016/j.system.2020.102352

[ref30] Mansfield C. F. Beltman S. Broadley T. Weatherby-Fell N. (2016). Building resilience in teacher education: an evidenced informed framework. Teach. Teach. Educ. 54, 77–87. doi: 10.1016/j.tate.2015.11.016

[ref31] Mansfield C. F. Beltman S. Price A. McConney A. (2012). “Don’t sweat the small stuff:” understanding teacher resilience at the chalkface. Teach. Teach. Educ. 28, 357–367. doi: 10.1016/j.tate.2011.11.001

[ref32] Masten A. S. Best K. M. Garmezy N. (1990). Resilience and development: contributions from the study of children who overcome adversity. Dev. Psychopathol. 2, 425–444. doi: 10.1017/s0954579400005812

[ref33] Mukadimah H. (2025). Exploring teacher resilience in Indonesia: a scoping review of challenges, strategies, and support mechanisms. Jurnal Ilmiah WUNY 7, 44–54. doi: 10.21831/jwuny.v7i2.84213

[ref34] Qu J. Wang M. Xin Y. (2025). The role of resilience and perseverance of effort among Chinese EFL teachers’ work engagement. Porta Linguarum Revista Interuniversitaria de Didáctica de Las Lenguas Extranjeras 43, 11–28. doi: 10.30827/portalin.vi43.28309

[ref35] Rezazadeh K. Janebi Enayat M. Poorebrahim F. (2023). Exploring bilingual EFL teacher resilience in the Iranian non-profit and state schools: a mixed-methods study. Asian-Pac. J. Second Foreign Lang. Educ. 8. doi: 10.1186/s40862-023-00196-3

[ref36] Richardson G. E. Neiger B. L. Jensen S. Kumpfer K. L. (1990). The resiliency model. Health Educ. 21, 33–39. doi: 10.1080/00970050.1990.10614589

[ref37] Rogers G. Szomszor M. Adams J. (2020). Sample size in bibliometric analysis. Scientometrics 125, 777–794. doi: 10.1007/s11192-020-03647-7

[ref38] Schwarze J. Wosnitza M. (2018). “How does apprentice resilience work?” in Resilience in Education: Concepts, Contexts and Connections, eds. Wosnitza M. Peixoto F. Beltman S. Mansfield C. F. (Cham: Springer International Publishing), 35–52.

[ref39] Seligman M. E. Csikszentmihalyi M. (2000). Positive psychology: an introduction. Am. Psychol. 55, 5–14. doi: 10.1037//0003-066x.55.1.5, 11392865

[ref40] Shirazizadeh M. Abbaszadeh A. (2023). EFL teacher resilience: instrument development and validation. Refl. Pract. 24, 375–388. doi: 10.1080/14623943.2023.2200926

[ref41] Wang Y. Derakhshan A. Rahimpour H. (2024). Developing resilience among Chinese and Iranian EFL teachers: a multi-dimensional cross-cultural study. J. Multiling. Multicult. Dev. 45, 2111–2128. doi: 10.1080/01434632.2022.2042540

[ref42] Wang Y. Derakhshan A. Zhang L. J. (2021). Researching and practicing positive psychology in second/foreign language learning and teaching: the past, current status and future directions. Front. Psychol. 12:731721. doi: 10.3389/fpsyg.2021.731721, 34489835 PMC8417049

[ref43] Wang X. Gao Y. Wang Q. Zhang P. (2024). Relationships between self-efficacy and teachers’ well-being in middle school English teachers: the mediating role of teaching satisfaction and resilience. Behav. Sci. 14:629. doi: 10.3390/bs14080629, 39199025 PMC11351107

[ref44] Xie F. (2021). A study on Chinese EFL teachers’ work engagement: the predictability power of emotion regulation and teacher resilience. Front. Psychol. 12:735969. doi: 10.3389/fpsyg.2021.735969, 34512487 PMC8430242

[ref45] Zhang L. (2023). Reviewing the effect of teachers’ resilience and wellbeing on their foreign language teaching enjoyment. Front. Psychol. 14:1187468. doi: 10.3389/fpsyg.2023.1187468, 37720655 PMC10501855

[ref46] Zhi R. Derakhshan A. (2024). Modelling the interplay between resilience, emotion regulation and psychological well-being among Chinese English language teachers: the mediating role of self-efficacy beliefs. Eur. J. Educ. 59:e12643. doi: 10.1111/ejed.12643

